# Development of an unbiased statistical method for the analysis of unigenic evolution

**DOI:** 10.1186/1471-2105-7-150

**Published:** 2006-03-17

**Authors:** Colleen D Behrsin, Chris J Brandl, David W Litchfield, Brian H Shilton, Lindi M Wahl

**Affiliations:** 1 Department of Biochemistry, University of Western Ontario, London, Ontario, Canada; 2 Department of Applied Mathematics, University of Western Ontario, London, Ontario, Canada

## Abstract

**Background:**

Unigenic evolution is a powerful genetic strategy involving random mutagenesis of a single gene product to delineate functionally important domains of a protein. This method involves selection of variants of the protein which retain function, followed by statistical analysis comparing expected and observed mutation frequencies of each residue. Resultant mutability indices for each residue are averaged across a specified window of codons to identify hypomutable regions of the protein. As originally described, the effect of changes to the length of this averaging window was not fully eludicated. In addition, it was unclear when sufficient functional variants had been examined to conclude that residues conserved in all variants have important functional roles.

**Results:**

We demonstrate that the length of averaging window dramatically affects identification of individual hypomutable regions and delineation of region boundaries. Accordingly, we devised a region-independent chi-square analysis that eliminates loss of information incurred during window averaging and removes the arbitrary assignment of window length. We also present a method to estimate the probability that conserved residues have not been mutated simply by chance. In addition, we describe an improved estimation of the expected mutation frequency.

**Conclusion:**

Overall, these methods significantly extend the analysis of unigenic evolution data over existing methods to allow comprehensive, unbiased identification of domains and possibly even individual residues that are essential for protein function.

## Background

The completion of genome sequencing projects has led to the identification of novel proteins at an unprecedented rate [1-4]. In many cases, sequence similarities with previously characterized proteins yield obvious insights into function. By comparison, many novel proteins fail to exhibit significant similarity to other proteins or exhibit similarity only to proteins of unknown activity. Even in cases where proteins exhibit extensive conservation with homologues of known biological function, their actions may remain poorly defined because the specific domains required for function are unclear.

One innovative experimental approach with the capacity to identify domains and possibly even specific amino acid residues that are required for function is a genetic strategy known as unigenic evolution, developed by Deminoff *et al *[5]. Unigenic evolution involves random mutagenesis of a gene whose loss gives rise to a selectable phenotype [5-9]. Randomly mutagenized variants of the gene that retain function are subsequently isolated and characterized by DNA sequencing. An underlying assumption for the unigenic evolution strategy is that regions of the protein that are required for function will be conserved whereas regions that are dispensable for function will be extensively mutated in variants that retain function. However, by itself, this selection does not exclude the possibility that missense mutations within specific domains or residues are infrequently observed simply because of differences in transition and transversion rates, or due to the degeneracy of the genetic code. To address this issue, Deminoff *et al*.[5], developed a statistical analysis that involves comparison of the expected frequency of mutation to the observed frequency of mutation for each residue. To increase statistical power, the calculated mutability indices for individual residues are averaged using a sliding window of a pre-defined length. This procedure allows putative hypomutable regions within the protein to be identified by visual inspection. The statistical significance of each putative region is then determined by computing χ^2 ^.

The results of the statistical analysis described by Deminoff *et al*.[5] depend on the number of residues that are averaged in calculating mutability, that is, on the length of the sliding window, which is chosen arbitrarily. We have therefore developed a region-independent chi-square analysis to improve the identification of hypomutable regions. Since not all transitions and transversions were equally likely in our laboratory, we also refined the calculation of the expected frequency of mutation to include each base-to-base mutation rate. Finally, we extended the analysis of Deminoff *et al*.[5] to address an experimentally critical question of whether an individual residue has not been mutated simply because an insufficient population of mutated variants has been analyzed. Collectively, these advances provide for the unbiased identification of hypomutable regions and for assessing the confidence levels for individual hypomutable regions or conserved residues, based on the number of functional variants that have been analysed. We illustrate our technique using data generated by the unigenic evolution of the human peptidyl-prolyl isomerase Pin1 [8,10,11].

## Results

Since the goal of unigenic evolution is to identify residues that are critical to protein function [5-9], we focus our attention on residues for which no missense amino acid substitutions are observed in any of the sequenced functional molecules. Rather than conveying functional importance, some of these non-mutated residues may represent codons for which missense mutations are intrinsically less likely, due to the degeneracy of the genetic code. To distinguish between these possibilities, we must assess the inherent mutability of each residue within the protein. In a later section, we will consider another important possibility: that some residues have remained non-mutated in all the functional sequences simply by chance.

Since the mutational data generated by unigenic evolution contains both missense and silent nucleotide substitutions, an observed frequency of missense mutations (*f*_obs. missense_) for each codon in the protein can be calculated. Following Deminoff *et al*. [5] this frequency is defined as the number of missense mutations divided by the total number of mutations (missense plus silent):

fobs. missense = # missense mutations# missense mutations + # silent mutations     (1)
 MathType@MTEF@5@5@+=feaafiart1ev1aaatCvAUfKttLearuWrP9MDH5MBPbIqV92AaeXatLxBI9gBaebbnrfifHhDYfgasaacH8akY=wiFfYdH8Gipec8Eeeu0xXdbba9frFj0=OqFfea0dXdd9vqai=hGuQ8kuc9pgc9s8qqaq=dirpe0xb9q8qiLsFr0=vr0=vr0dc8meaabaqaciaacaGaaeqabaqabeGadaaakeaacqWGMbGzdaWgaaWcbaGaee4Ba8MaeeOyaiMaee4CamNaeeOla4IaeeiiaaIaeeyBa0MaeeyAaKMaee4CamNaee4CamNaeeyzauMaeeOBa4Maee4CamNaeeyzaugabeaakiabbccaGiabb2da9iabbccaGmaalaaabaGaee4iamIaeeiiaaIaeeyBa0MaeeyAaKMaee4CamNaee4CamNaeeyzauMaeeOBa4Maee4CamNaeeyzauMaeeiiaaIaeeyBa0MaeeyDauNaeeiDaqNaeeyyaeMaeeiDaqNaeeyAaKMaee4Ba8MaeeOBa4Maee4CamhabaGaee4iamIaeeiiaaIaeeyBa0MaeeyAaKMaee4CamNaee4CamNaeeyzauMaeeOBa4Maee4CamNaeeyzauMaeeiiaaIaeeyBa0MaeeyDauNaeeiDaqNaeeyyaeMaeeiDaqNaeeyAaKMaee4Ba8MaeeOBa4Maee4CamNaeeiiaaIaee4kaSIaeeiiaaIaee4iamIaeeiiaaIaee4CamNaeeyAaKMaeeiBaWMaeeyzauMaeeOBa4MaeeiDaqNaeeiiaaIaeeyBa0MaeeyDauNaeeiDaqNaeeyyaeMaeeiDaqNaeeyAaKMaee4Ba8MaeeOBa4Maee4CamhaaiaaxMaacaWLjaWaaeWaaeaacqaIXaqmaiaawIcacaGLPaaaaaa@92C5@

This observed frequency of missense mutations can then be compared to the expected frequency of missense mutations for each corresponding codon. The expected frequency of missense mutation is calculated by observing that each codon has a characteristic potential for producing a silent or missense mutation given one nucleotide change. Looking at all possible single base changes, the expected frequency of missense mutations can be easily calculated. This "first pass" technique assumes that all single nucleotide substitutions are equally likely.

Deminoff *et al.*[5] improved this technique by correcting for a significant bias in the mutation frequencies in unigenic evolution data; in particular, in their study they noted that 80% of observed base changes were transitions and 20% transversions. This is largely a consequence of the fact that errors made by the mutagenic agent, Taq DNA polymerase, are heavily biased toward purine to purine and pyrimidine to pyrimidine base changes. We have observed a very similar ratio (79:21) in the functional variants characterized in our experimental work [8]. A more accurate value of *f*_exp. missense _for each codon can therefore be calculated by determining the frequencies of missense substitutions created by either transitions or transversions, and weighting these expectations by the observed frequency of transitions and transversions in the database [5].

Normalizing the frequency of expected missense mutations (based on codon sequence) to the transition/transversion ratios observed in functional clones greatly improves the accuracy of the expected value. However, this technique assumes i) that all transitions (or all transversions) are equally likely and ii) that substitutions observed in functional clones are representative of all substitutions that occur in unigenic evolution. Since selection for viable mutants results in a bias toward mutations that are tolerated by functional molecules, we expect that each of these assumptions may result in some substitution frequencies that are over- or under-represented. We therefore extended the analysis presented in Deminoff *et al*. [5] to take into account the individual substitution frequencies incurred during unigenic evolution in our hands, and evaluated the extent to which these two assumptions hold.

### Mutation frequencies from a random pool

To remove any possible bias caused by considering only the mutations in functional clones, we analyzed a stratified random sample of protein clones (6 clones from each of three mutant libraries), where the libraries contained all functional and non-functional clones. We then determined the frequency of each substitution for each nucleotide in this random pool. The distribution of observed nucleotide substitutions in the random pool is given in Table 1. Note that the transition/transversion ratio observed in this dataset was 74:26, in contrast to the 79:21 ratio observed in functional clones [8]. Furthermore, the transition/transversion ratio for adenine was 65.5:35.5 providing evidence that all possible transitions and all transversions are not equally likely.

Using the nucleotide substitution data that was observed within this random sample of clones, equations were formulated to calculate the expected frequency of missense mutations (*f*_exp. missense_) for each codon. The first step in this analysis is to estimate the underlying mutation rates for each base. The probability that a base, B, is replaced by substitution in one run through PCR is defined as mB. Since the substitution may actually have occured on the complementary strand, we treat a mutation from A to G, for example, as equivalent to a substitution from T to C. Thus the probability of mutating an adenine to any other nucleotide is given by:

mA=(#A-G) + (#T-C) + (#A-C) + (#T-G) + (#A-T) + (#T-A)(Total # of A's + total # of T's in sequence)(# of Random Clones Sequenced)
 MathType@MTEF@5@5@+=feaafiart1ev1aaatCvAUfKttLearuWrP9MDH5MBPbIqV92AaeXatLxBI9gBaebbnrfifHhDYfgasaacH8akY=wiFfYdH8Gipec8Eeeu0xXdbba9frFj0=OqFfea0dXdd9vqai=hGuQ8kuc9pgc9s8qqaq=dirpe0xb9q8qiLsFr0=vr0=vr0dc8meaabaqaciaacaGaaeqabaqabeGadaaakeaacqqGTbqBdaWgaaWcbaGaeeyqaeeabeaakiabg2da9maalaaabaGaeiikaGIaee4iamIaeeyqaeKaeeyla0Iaee4raCKaeeykaKIaeeiiaaIaee4kaSIaeeiiaaIaeeikaGIaee4iamIaeeivaqLaeeyla0Iaee4qamKaeeykaKIaeeiiaaIaee4kaSIaeeiiaaIaeeikaGIaee4iamIaeeyqaeKaeeyla0Iaee4qamKaeeykaKIaeeiiaaIaee4kaSIaeeiiaaIaeeikaGIaee4iamIaeeivaqLaeeyla0Iaee4raCKaeeykaKIaeeiiaaIaee4kaSIaeeiiaaIaeeikaGIaee4iamIaeeyqaeKaeeyla0IaeeivaqLaeeykaKIaeeiiaaIaee4kaSIaeeiiaaIaeeikaGIaee4iamIaeeivaqLaeeyla0IaeeyqaeKaeeykaKcabaGaeeikaGIaeeivaqLaee4Ba8MaeeiDaqNaeeyyaeMaeeiBaWMaeeiiaaIaee4iamIaeeiiaaIaee4Ba8MaeeOzayMaeeiiaaIaeeyqaeKaee4jaCIaee4CamNaeeiiaaIaee4kaSIaeeiiaaIaeeiDaqNaee4Ba8MaeeiDaqNaeeyyaeMaeeiBaWMaeeiiaaIaee4iamIaeeiiaaIaee4Ba8MaeeOzayMaeeiiaaIaeeivaqLaee4jaCIaee4CamNaeeiiaaIaeeyAaKMaeeOBa4MaeeiiaaIaee4CamNaeeyzauMaeeyCaeNaeeyDauNaeeyzauMaeeOBa4Maee4yamMaeeyzauMaeeykaKIaeeikaGIaee4iamIaeeiiaaIaee4Ba8MaeeOzayMaeeiiaaIaeeOuaiLaeeyyaeMaeeOBa4MaeeizaqMaee4Ba8MaeeyBa0MaeeiiaaIaee4qamKaeeiBaWMaee4Ba8MaeeOBa4MaeeyzauMaee4CamNaeeiiaaIaee4uamLaeeyzauMaeeyCaeNaeeyDauNaeeyzauMaeeOBa4Maee4yamMaeeyzauMaeeizaqMaeeykaKcaaaaa@B54F@

where (#A-G) represents the number of A to G substitutions observed in the random pool of functional and non-functional clones (Table 1).

Similarly, probabilities for individual nucleotide substitutions (denoted m_A-G_, m_A-C_, etc.) can by calculated using the same mutational data from the random pool. As a sample dataset, a summary of the nucleotide substitution rates observed in the random pool of clones is given in Table 2.

Based on these mutation probabilities, the frequency of expected missense substitutions for each codon can be calculated. An amino acid with more than one codon such as Cys (TGC and TGT) will exhibit distinct *f*_exp. missense _values for each codon. For example, the frequency of expected missense substitutions for Cys (TGC) was calculated using the following equation:

fexp. missense Cys (TGC) = (mT+mG+[mC-G+mC-A])(mT+mG+mC)
 MathType@MTEF@5@5@+=feaafiart1ev1aaatCvAUfKttLearuWrP9MDH5MBPbIqV92AaeXatLxBI9gBaebbnrfifHhDYfgasaacH8akY=wiFfYdH8Gipec8Eeeu0xXdbba9frFj0=OqFfea0dXdd9vqai=hGuQ8kuc9pgc9s8qqaq=dirpe0xb9q8qiLsFr0=vr0=vr0dc8meaabaqaciaacaGaaeqabaqabeGadaaakeaacqWGMbGzdaWgaaWcbaGaeeyzauMaeeiEaGNaeeiCaaNaeeOla4IaeeiiaaIaeeyBa0MaeeyAaKMaee4CamNaee4CamNaeeyzauMaeeOBa4Maee4CamNaeeyzaugabeaakiabbccaGiabboeadjabbMha5jabbohaZjabbccaGiabbIcaOiabbsfaujabbEeahjabboeadjabbMcaPiabbccaGiabb2da9iabbccaGmaalaaabaGaeeikaGIaeeyBa02aaSbaaSqaaiabbsfaubqabaGccqGHRaWkcqqGTbqBdaWgaaWcbaGaee4raCeabeaakiabgUcaRiabcUfaBjabb2gaTnaaBaaaleaacqqGdbWqcqqGTaqlcqqGhbWraeqaaOGaey4kaSIaeeyBa02aaSbaaSqaaiabboeadjabb2caTiabbgeabbqabaGccqGGDbqxcqGGPaqkaeaacqGGOaakcqqGTbqBdaWgaaWcbaGaeeivaqfabeaakiabgUcaRiabb2gaTnaaBaaaleaacqqGhbWraeqaaOGaey4kaSIaeeyBa02aaSbaaSqaaiabboeadbqabaGccqGGPaqkaaaaaa@6D34@

Note that it is possible that some missense mutations may actually be nonsense (e.g. TGA). To avoid *a priori *assumptions about whether nonsense mutations would be deleterious, we did not exclude these from the frequency of expected missense mutations calculated above.

Again, as a sample dataset, calculated values of *f*_exp. missense _for each codon in Pin1 are given in Table 3; corresponding values of the transition/transversion normalized mutation rates from the functional pool of molecules as determined by Deminoff *et al*.[5] are shown for comparison. Although values for some codons are similar, in most cases results obtained by the two methods differ by 5% or more. This issue will be taken up again in the Conclusions.

### Identifying hypomutable regions

The observed frequency of missense mutations for each codon in the protein calculated from equation (1) can be compared to the expected frequency (Table 3) to determine the mutability of each residue. When the observed missense frequency is less than our expected value, we use the standard measure:

H=(fobs. missense−fexp. missense)fexp. missense
 MathType@MTEF@5@5@+=feaafiart1ev1aaatCvAUfKttLearuWrP9MDH5MBPbIqV92AaeXatLxBI9gBaebbnrfifHhDYfgasaacH8akY=wiFfYdH8Gipec8Eeeu0xXdbba9frFj0=OqFfea0dXdd9vqai=hGuQ8kuc9pgc9s8qqaq=dirpe0xb9q8qiLsFr0=vr0=vr0dc8meaabaqaciaacaGaaeqabaqabeGadaaakeaacqqGibascqGH9aqpdaWcaaqaaiabcIcaOiabdAgaMnaaBaaaleaacqqGVbWBcqqGIbGycqqGZbWCcqqGUaGlcqqGGaaicqqGTbqBcqqGPbqAcqqGZbWCcqqGZbWCcqqGLbqzcqqGUbGBcqqGZbWCcqqGLbqzaeqaaOGaeyOeI0IaemOzay2aaSbaaSqaaiabbwgaLjabbIha4jabbchaWjabb6caUiabbccaGiabb2gaTjabbMgaPjabbohaZjabbohaZjabbwgaLjabb6gaUjabbohaZjabbwgaLbqabaGccqGGPaqkaeaacqWGMbGzdaWgaaWcbaGaeeyzauMaeeiEaGNaeeiCaaNaeeOla4IaeeiiaaIaeeyBa0MaeeyAaKMaee4CamNaee4CamNaeeyzauMaeeOBa4Maee4CamNaeeyzaugabeaaaaaaaa@689A@

to determine the hypomutability of the residue. The value of H will range between 0 and -1, where -1 reflects maximal hypomutability and occurs when we observe zero missense mutations; zero occurs when the observed frequency equals the expected frequency.

When the observed missense frequency is greater than the expected value, however, H ranges between zero and M = (1-*f*_exp. missense_)/*f*_exp. missense_. Since M is a (possibly large) number that varies from one codon to the next, we normalize hypermutability in this case by dividing H by the theoretical maximum, M, for that residue. This normalized hypermutability has a minimum of zero and a maximum value of +1, which only occurs if all mutations observed are missense mutations. To plot these results, the mutability of individual residues is averaged over a window of a specified number of codons. The average hypo- or hypermutability is then plotted in the center of the specified window, and the window is shifted downstream one codon at a time. Note that this normalization and plotting technique, although described in different terms, is equivalent to the method previously described by Deminoff *et al*.[5].

**Figure 1:**
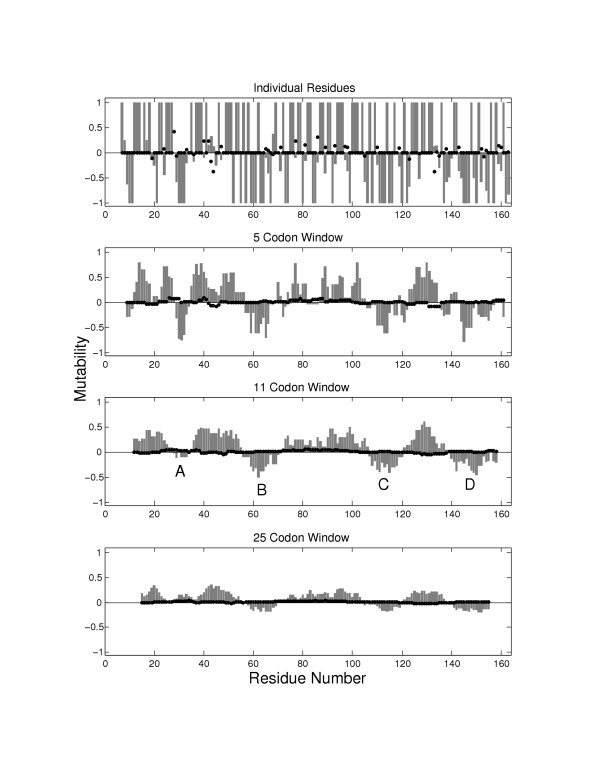
**Mutability plots**. Mutability plots were determined as described in the text (grey bars). The mutability of individual residues was averaged over a window of 1, 5, 11 or 25 codons. The hypo- or hypermutability was then plotted as a bar in the center of the specified window and the window was shifted downstream one codon at a time. Individual hypomutable regions, designated A, B, C, and D are indicated on the plot for the 11 codon window. For comparison, the difference between mutability calculated by previous methods (5) and mutability as described in this manuscript is also shown (circles).

Since no objective means for choosing the length of the averaging window have been established, we investigated the dependence of our results on this length. Accordingly, we applied the procedure described above with window lengths ranging from 1–25 codons to the sample dataset (Figure 1). At one extreme, the window length of one produces a plot of hypo-and hypermutable residues, as opposed to regions. However, as discussed in a later section, the reliability of this latter plot is questionable since our data set does not contain sufficient information to generate statistically significant mutation frequencies for each residue. Increasing the window size between 5 and 25 codons decreases the number of hypomutable regions; using a 5-codon window at least 5 discrete hypomutable regions are apparent, while a 25-codon window reveals only 3 discrete hypomutable regions. Since delineation of the boundaries of each hypomutable region using the strategy of Deminoff *et al*.[5] depends on the initial identification of hypomutable regions by visual inspection, it is clear from this example that window length can have a dramatic influence on the analysis.

### Determining region boundaries and significance

In addition to the influence of the window size on the number of hypomutable regions, it is important to recognize that the boundaries of each hypomutable region are not always clearly defined. To determine the significance of putative hypomutable regions and to define their boundaries statistically, a chi-square (χ^2^) analysis of each region is performed. For a series of residues corresponding to each apparently hypomutable region, χ^2 ^can be determined using the expression:

χ2=(abs [total # observed missense - total # expected missense ] - 0.5)2total # expected missense+(abs [total # observed silent - total # expected silent] - 0.5)2total # expected silent
 MathType@MTEF@5@5@+=feaafiart1ev1aaatCvAUfKttLearuWrP9MDH5MBPbIqV92AaeXatLxBI9gBaebbnrfifHhDYfgasaacH8akY=wiFfYdH8Gipec8Eeeu0xXdbba9frFj0=OqFfea0dXdd9vqai=hGuQ8kuc9pgc9s8qqaq=dirpe0xb9q8qiLsFr0=vr0=vr0dc8meaabaqaciaacaGaaeqabaqabeGadaaakqaaceqaaiabeE8aJnaaCaaaleqabaGaeGOmaidaaOGaeyypa0ZaaSaaaeaacqGGOaakcqqGHbqycqqGIbGycqqGZbWCcqqGGaaicqqGBbWwcqqG0baDcqqGVbWBcqqG0baDcqqGHbqycqqGSbaBcqqGGaaicqqGJaWicqqGGaaicqqGVbWBcqqGIbGycqqGZbWCcqqGLbqzcqqGYbGCcqqG2bGDcqqGLbqzcqqGKbazcqqGGaaicqqGTbqBcqqGPbqAcqqGZbWCcqqGZbWCcqqGLbqzcqqGUbGBcqqGZbWCcqqGLbqzcqqGGaaicqqGTaqlcqqGGaaicqqG0baDcqqGVbWBcqqG0baDcqqGHbqycqqGSbaBcqqGGaaicqqGJaWicqqGGaaicqqGLbqzcqqG4baEcqqGWbaCcqqGLbqzcqqGJbWycqqG0baDcqqGLbqzcqqGKbazcqqGGaaicqqGTbqBcqqGPbqAcqqGZbWCcqqGZbWCcqqGLbqzcqqGUbGBcqqGZbWCcqqGLbqzcqqGGaaicqqGDbqxcqqGGaaicqqGTaqlcqqGGaaicqqGWaamcqqGUaGlcqqG1aqncqqGPaqkdaahaaWcbeqaaiabbkdaYaaaaOqaaiabbsha0jabb+gaVjabbsha0jabbggaHjabbYgaSjabbccaGiabbocaJiabbccaGiabbwgaLjabbIha4jabbchaWjabbwgaLjabbogaJjabbsha0jabbwgaLjabbsgaKjabbccaGiabb2gaTjabbMgaPjabbohaZjabbohaZjabbwgaLjabb6gaUjabbohaZjabbwgaLbaaaeaacqGHRaWkdaWcaaqaaiabcIcaOiabbggaHjabbkgaIjabbohaZjabbccaGiabbUfaBjabbsha0jabb+gaVjabbsha0jabbggaHjabbYgaSjabbccaGiabbocaJiabbccaGiabb+gaVjabbkgaIjabbohaZjabbwgaLjabbkhaYjabbAha2jabbwgaLjabbsgaKjabbccaGiabbohaZjabbMgaPjabbYgaSjabbwgaLjabb6gaUjabbsha0jabbccaGiabb2caTiabbccaGiabbsha0jabb+gaVjabbsha0jabbggaHjabbYgaSjabbccaGiabbocaJiabbccaGiabbwgaLjabbIha4jabbchaWjabbwgaLjabbogaJjabbsha0jabbwgaLjabbsgaKjabbccaGiabbohaZjabbMgaPjabbYgaSjabbwgaLjabb6gaUjabbsha0jabb2faDjabbccaGiabb2caTiabbccaGiabbcdaWiabb6caUiabbwda1iabbMcaPmaaCaaaleqabaGaeeOmaidaaaGcbaGaeeiDaqNaee4Ba8MaeeiDaqNaeeyyaeMaeeiBaWMaeeiiaaIaee4iamIaeeiiaaIaeeyzauMaeeiEaGNaeeiCaaNaeeyzauMaee4yamMaeeiDaqNaeeyzauMaeeizaqMaeeiiaaIaee4CamNaeeyAaKMaeeiBaWMaeeyzauMaeeOBa4MaeeiDaqhaaaaaaa@0D47@

In this equation, the total number of expected missense mutations for each hypomutable region is calculated by multiplying the total number of mutations (silent + missense) observed in a hypomutable region by the average value of *f*_*exp. missense *_for all codons within the region; the expected number of silent mutations in the region is calculated similarly. The Yates correction has been used [5,12] since the outcomes fall into only two classes: silent or missense mutations. The *P *value for each hypomutable region can then be evaluated for one degree of freedom.

Deminoff *et al*. [5] suggest considering a subset of residues in the center of each putative hypomutable region and gradually expanding the boundaries, calculating a new χ^2 ^value for each series of residues until *P *falls below significance. Using this technique, and identifying putative regions based on the graph obtained with an 11-codon window for the Pin1 data, we identified four significantly hypomutable regions with the following levels of significance: region A, *P <*0.0025; region B, *P *< 0.001; region C, *P *< 0.1; region D, *P *< 0.0025.

Using this technique, however, we found that delineation of the boundaries of each region was somewhat arbitrary. For example, a region of 8 residues might be significant. If the 9^th ^residue is added to the region, significance is lost. However if both the 9^th ^and 10^th ^residues are added, significance is regained. Similarly, we found that region boundaries were sensitive to the initial choice of "central" residues, and to the direction in which the region was first expanded.

We therefore developed a region-independent method for identifying significant hypomutable regions. In this method, we consider region lengths up to 50 residues long. For each region length, we calculate χ^2 ^for every possible region of that length in our sequence. This corresponds once again to sliding a window of the appropriate length along the sequence, however in this case we are not averaging across the window, but computing the significance of the region within the window as a whole. Thus, for example, computing the χ^2 ^value for an 11-codon region is not equivalent to computing the average hypomutability in a window of the same length.

**Figure 2:**
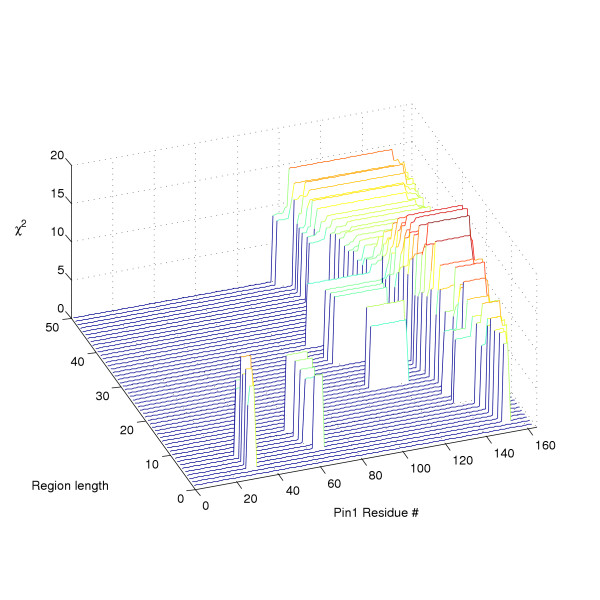
**Region-independent chi-square analysis**. Three-dimensional plot illustrating χ^2 ^of each significantly hypomutable region plotted against region length and amino acid residue number. Calculations were performed as described in the text. Only regions that are significant at the 0.005 level are plotted; the whole window is plotted whenever this significance level is achieved. If a residue is involved in more than one significant region of the same length, the region with the highest χ^2 ^value is plotted. Colours indicate χ^2 ^of the region and range from deep red (χ^2 ^> 15, corresponding to α < 0.0001) to pale green (χ^2 ^> 8, α < 0.005). Four hypomutable regions approximating regions A-D (Figure 1) are evident.

With these values in hand, we produce a 3-dimensional plot of the χ^2 ^value for each region of every length (Figure 2); the region length and the residues which constitute the region give the two independent axes. We plot the entire region for all regions whose χ^2 ^value exceeded the *α *= 0.005 significance level. This unusually strict level of significance was used to correct for multiple significance testing, as described below. Note that χ^2 ^does not distinguish between significantly hypo- or hypermutable regions, thus we only plot χ^2 ^for significantly hypomutable regions (i.e. if the observed number of missense mutations is less than expected). The figure reveals four hypomutable regions, corresponding relatively closely to regions A through D described above. We find that region A is only significant for fairly short region lengths. Region B is significant over a wider range of lengths, and the χ^2 ^value associated with the region changes depending on region length. In region C, we observe the effect described previously: regions of length 17 and 18 are significant, but significance is lost for regions of length 19 through 24. If the region is expanded to lengths of 25 through 27, however, significance is regained. Region D is significant for almost any region length, reaching its highest χ^2 ^values for region lengths of about 25 residues.

Although a plot such as Figure 2 does not completely remove the arbitrariness in determining region boundaries, it greatly clarifies decisions regarding which regions to consider in further biochemical analyses. Furthermore, this strategy ensures that no statistically-significant hypomutable regions are missed because an inappropriate window length has been used. Due to multiple significance testing, however, we expect some regions to appear significant simply by chance. Although a Bonferroni correction is not appropriate for the highly non-independent tests illustrated here, we can instead estimate the expected number of such false positives. A conservative approach is to consider the number of non-overlapping (and thus independent) regions tested in each row of the figure. For a window length of one codon, we have 164 independent regions and at *α *= 0.005, we expect on average 0.8 false positives. The false positive rate falls rapidly as the window length increases, however. For a window length of three codons, we have 164/3 non-overlapping regions and expect 0.27 false positives. Thus there is about a 1 in 4 chance that each of the two significant regions illustrated in Figure 2 for region length 3 appears to be hypomutable only by chance. The appropriate level of tolerance for false positives will clearly differ depending on the experimental protocol; we recommend that the value of *α *be chosen accordingly.

### Significance of non-mutated codons

As stated previously, the observed hypomutability of residues or regions within a protein could result from selection against mutations located within essential regions, through differences in the mutation frequency of various codons, or simply by chance. The overall goal of unigenic evolution is to identify residues for which the first of these factors is important [5]. The analysis in the previous sections is designed to normalize for the second of these factors, variable mutation rates. This leaves us with one further question: for a specific codon in the protein, what is the probability a missense substitution never occurred at this codon simply by chance? When this probability is sufficiently low for all codons in the protein, we can conclude with some confidence that we have sequenced "enough" functional molecules – enough, that is, to draw statistically meaningful conclusions about those codons which remain conserved. In order to answer this question we consider each codon in the protein in turn, and calculate *Q*: the probability that in all sequenced functional clones, a missense substitution was never observed at this codon simply because not enough functional clones were sequenced. We use lowercase *q *to denote the per clone "quality" factor: the probability that a missense mutation did not occur at a specific codon following a single round of PCR-mediated mutagenesis. If *F *functional clones were sequenced in total, *Q *is then given by *Q *= *q*^F^. Thus *q *is the probability that mutagenesis missed this codon by chance in one functional clone, and *Q *is the probability that mutagenesis missed this codon in *F *functional clones.

As with the expected mutation rates calculated in previous sections, *q *values should be calculated using data obtained from a random sample of mutant clones, including both functional and non-functional sequences. Given such data, we determine the probability that each codon is conserved during a single run through the PCR using our estimates of the underlying mutation rates. For example, to calculate the probability that a methionine (ATG) residue was conserved in a single run through PCR, we evaluate:

*q*_Met _= (1- m_A_)(1- m_T_)(1- m_G_)

For an amino acid with multiple codons such as cysteine (TGC and TGT) *q *values are determined as follows:

*q*_Cys (TGC) _= (1- m_T_)(1- m_G_) (1- m_C-G _+ m_C-A_)

The latter equation follows from the fact that a mutation of the first two nucleotides (T and G) to any other nucleotide results in an amino acid change, while a mutation of the third base results in a missense substitution only if mutated to a G or A.

Once again we provide a sample dataset from the unigenic evolution of Pin1 in Table 4. The table gives the calculated *Q *values for each of the 39 residues from positions 7–163 in Pin1 that were not mutated by unigenic evolution. (Residues 1–6 were not listed as they were covered by the forward primer in PCR mutagenesis.) Fifteen of the 39 conserved residues in this example have *Q *values > 0.05. This implies there was more than a 5% probability that no mutations occurred at these residues simply because not enough functional molecules had been analyzed to observe a change.

For the 24 residues with *Q *< 0.05, we must also take into account the issue of multiple significance testing. The average value of *Q *for these residues is 0.0182. Thus there is on average less than a 2% chance that mutagenesis missed each of these codons. Overall the expected number of false positives is therefore 0.0182*24 = 0.44, or less than one of the 24 residues.

## Discussion

Building on previous analytical work in this area [5], we set out to present a detailed description of data analysis for unigenic evolution, including new statistical considerations and relaxing some of the limiting assumptions. An experimental data set was used to evaluate the extent to which results obtained by our method differ from those generated by previous techniques.

To refine the analysis of Deminoff *et al*. [5] who based the frequency of expected mutations on the transition and transversion frequencies observed in functional clones, we used a random pool of both functional and non-functional clones to estimate the underlying base-to-base substitution rates in our experimental protocol. As expected, the number of nucleotide substitutions observed within the random pool of 18 clones was nearly double that within the 83 functional clones, demonstrating that selection for functional clones eliminated most clones that were subject to a large number of amino acid changes. Furthermore, it would be expected that certain amino acid substitutions would be more or less frequent in functional clones.

Comparison of *f*_exp. missense _values for all codons in our sample data set revealed that this was indeed the case (Table 3). Although several codons exhibited similar expected mutation frequencies when calculated by both methods, the *f*_exp. missense _values of most codons differed. Specifically, the expected frequency of missense mutations differed by 5% or more for 31 of 42 codons. This indicates that mutation rates in the population of functional clones were not necessarily representative of mutation rates within the entire library of mutant alleles (both functional and non-functional).

Interestingly, an initial comparison of mutability plots generated using data from the random pool of clones (this study) to a plot generated using the transition/transversion ratio in functional clones [5] produced plots with similar overall patterns of mutability (see Figure 1). However, a chi-square analysis of the four hypomutable regions revealed that the *P *values of hypomutable regions A, B, and D were strikingly more significant when the *f*_exp. missense _values were calculated using data from the random pool. The *P *values generated using base-to-base mutations in the random pool were: region *A P*<0.0025; region B, *P *< 0.001; region C *P *< 0.1; region D, *P *< 0.0025. As a comparison, *P *values generated using transitions/transversions in functional clones were: region A *P *< 0.005; region B, *P *< 0.05, region C, *P *< 0.1; region D *P *< 0.05.

The data presented in Figure 1 also illustrate the influence of the size of the averaging window on the identification of hypomutable regions and consequently the delineation of the boundaries of these regions. Our analysis clearly illustrates that an averaging window that is too narrow (i.e. 5 codon) can result in the appearance of hypomutable regions that are not statistically significant, while an averaging window that is too broad (i.e. 25 codons) results in the disappearance of hypomutable regions that are in fact significant. By automating the process of the chi-square analysis over all possible region lengths, and by computing the significance of the region within the window as a whole without averaging, our strategy ensures that all statistically-significant hypomutable regions are identified.

We believe that another major practical contribution of this work is the derivation of *Q *= *q*^*F*^, where *Q *gives the probability that a missense mutation has not occurred at a particular codon by chance. As described above, when this probability is sufficiently low for all codons of interest in the protein, we can be reasonably certain that we have sequenced enough functional molecules to draw meaningful conclusions about those residues that remain conserved. We add the caveat that for some residues, sequencing "enough" functional molecules may not be feasible. As shown in Table 4, arginine (CGG) exhibited the highest *Q *value in our sample data set; the chance that missense substitutions were not observed at these non-mutated residues as a consequence of not analyzing enough functional clones exceeded 16%. This is a consequence of exceptionally low mutational frequencies, for example m_C-G _and m_G-C _in Table 2. Similar arguments can be applied to proline, alanine, and glycine codons. However, with the exception of these residues it was evident from analysis of the *Q *values that the probabilities of not observing missense substitutions simply by chance for the remaining 24 non-mutated residues were low. Thus, our method for identification of the boundaries of hypomutable regions facilitates additional "local" mutagenesis of these specific regions, for example by using random oligonucleotides, to further ensure that non-mutated residues are present because they are functionally critical.

Throughout this study, we used data obtained from the unigenic evolution of the peptidyl-prolyl isomerase Pin1 to illustrate our techniques. We used unigenic evolution in this case because the enzyme is highly conserved in eukaryotic organisms, and it was therefore difficult to identify functionally critical residues from a sequence alignment. The unigenic evolution strategy represents an unbiased approach that makes no a priori assumptions about which residues should be subjected to mutagenesis; furthermore, because residues other than alanine can be substituted in non-critical positions, new information about the amino acid chemistry required at each position is obtained. In the case of Pin1, unigenic evolution revealed four hypomutable regions, defined using the methods outlined in the current manuscript. Two of these functionally critical regions were subjected to saturating mutagenesis using random oligonucleotides, and functional clones were selected [8]. These experiments provided a more precise description of the functional importance of individual residues. For example, the crystal structure of Pin1 [17] revealed the presence of three residues, Lys63, Arg68, and Arg69 that participate in recognition of phosphorylated residues within the catalytic domain of Pin1. Although earlier studies had suggested that Arg68 and Arg69 are the two important residues within this region, our unigenic evolution analysis revealed that these residues were not conserved in all functional Pin1 clones. Instead, Lys63 was conserved with a very low Q value (see Table 4) suggesting that this residue is an essential residue for Pin1 function.

## Conclusion

Based on the results that we obtained in our experimental dataset, it can be readily envisaged that unigenic evolution together with the statistical methods that are described in this paper will be a powerful strategy for elucidating functional domains and, in some cases, specific residues that are essential for protein function.

## Methods

### Construction of libraries of Pin1 variants

Three independent libraries encoding variants of the human Pin1 cDNA [10] were generated using mutagenic PCR performed with Taq DNA polymerase as described in detail elsewhere [8]. Briefly, each of these independent libraries was constructed using 1 to 3 rounds of PCR, each consisting of 30 cycles. Due to the use of a primer encoding the first 6 amino acids of Pin1, base substitutions were incurred only in codons 7–163 of Pin1. In order to obtain sufficient statistical power, data from the three libraries were not analyzed separately.

### Isolation of functional Pin1 variants

Following mutagenesis, each of the three libraries was cloned into a yeast expression vector (pY204) to allow for selection of functional variants of Pin1 in the yeast strain YKH100 (*ess1 ::TRP1 *containing YCp88-*PIN1*) using a plasmid shuffling strategy [13]. Viability of this yeast strain which harbors a disruption of its Pin1 homolog *ESS1 *[14-16] is maintained by the human Pin1 cDNA that is expressed from a plasmid with a selectable *URA3 *marker. Following transformation of the Pin1 variant libraries into these yeast, functional variants were identified by their ability to support growth in the presence of 5-FOA [13]. Plasmids encoding functional variants of human Pin1 were isolated from yeast, transformed into bacteria and then isolated from bacteria for re-transformation into yeast. Plasmids that continued to support growth of yeast in the presence of 5-FOA upon transformation were subjected to DNA sequencing. A total of 83 functional Pin1 variants were isolated harboring a total of 460 nucleotide substitutions resulting in a total of 315 amino acid substitutions. A full description of the amino acid sequence of these variants is provided in Behrsin *et al *[8]. A total of 18 clones, representing 6 from each of the mutagenic Pin1 libraries, were randomly selected and analysed by DNA sequencing. The data sets derived from the characterization of the DNA sequences of the 83 functional Pin1 variants and the 18 random clones were used as a sample dataset for illustration of the proposed method.

## Authors' contributions

CDB carried out the experimental work for the unigenic evolution and participated in statistical analysis and drafting the manuscript. CJB, DWL and BHS jointly designed the experimental study and participated in improving the statistical analysis and drafting the manuscript. LMW designed the statistical method, participated in statistical analysis and drafted the manuscript. All authors have read and approved of the manuscript.

## Figures and Tables

**Table 1:** Nucleotide Substitutions in a Random Sample of 18 Unscreened Pin1 Clones.

**Nucleotide Substitution**	**# Observed**
A to G	38
T to C	35
	
G to A	25
C to T	28
	
T to A	11
A to T	16
	
C to A	8
G to T	5
	
A to C	4
T to G	0
	
C to G	0
G to C	1

**Table 2:** Estimated mutation probabilities based on 18 unscreened Pin1 clones.

**Nucleotide**	**Mutation Probability**
**m_A _and m_T_**	**0.0318**
m_A-C _and m_T-G_	0.0012
m_A-G _and m_T-C_	0.0223
m_A-T _and m_T-A_	0.0082

**m_C _and m_G_**	**0.0120**
m_C-A _and m_G-T_	0.0023
m_C-G _and m_G-C_	0.0002
m_C-T _and m_G-A_	0.0095

**Table 3:** Comparison of *f*_expected missense _values for Pin1 codons.

**Codon**	***f*_expected missense _this study**	***f*_expected missense _Deminoff et al.**
Met (ATG)	1.00	1.00
Trp (TGG)	1.00	1.00
**Cys (TGC)**	**0.83**	**0.76**
**Asp (GAC)**	**0.83**	**0.76**
**Asp (GAT)**	**0.70**	**0.76**
**Glu (GAA)**	**0.70**	**0.76**
**Glu (GAG)**	**0.83**	**0.76**
**Phe (TTC)**	**0.87**	**0.76**
Phe (TTT)	0.77	0.76
**His (CAC)**	**0.83**	**0.76**
Lys (AAA)	0.77	0.76
**Lys (AAG)**	**0.87**	**0.76**
**Asn (AAC)**	**0.87**	**0.76**
**Gln (CAG)**	**0.83**	**0.76**
**Tyr (TAC)**	**0.87**	**0.76**
**Ile (ATC)**	**0.90**	**0.72**
Ala (GCC)	0.67	0.67
Ala (GCG)	0.67	0.67
**Gly (GGA)**	**0.43**	**0.67**
Gly (GGC)	0.67	0.67
Gly (GGG)	0.67	0.67
**Gly (GGT)**	**0.43**	**0.67**
**Pro (CCA)**	**0.43**	**0.67**
Pro (CCC)	0.67	0.67
Pro (CCG)	0.67	0.67
**Pro (CCT)**	**0.43**	**0.67**
**Val (GTC)**	**0.78**	**0.67**
**Val (GTG)**	**0.78**	**0.67**
**Thr (ACC)**	**0.78**	**0.67**
**Thr (ACG)**	**0.78**	**0.67**
**Thr (ACT)**	**0.58**	**0.67**
**Ser (AGC)**	**0.83**	**0.76**
**Ser (AGT)**	**0.70**	**0.76**
**Ser (TCC)**	**0.78**	**0.67**
**Ser (TCT)**	**0.58**	**0.67**
**Ser (TCA)**	**0.58**	**0.67**
**Ser (TCG)**	**0.78**	**0.67**
**Leu (CTG)**	**0.61**	**0.43**
**Leu (CTC)**	**0.78**	**0.67**
Arg (AGA)	0.69	0.72
**Arg (AGG)**	**0.81**	**0.72**
**Arg (CGA)**	**0.39**	**0.62**

Boldface indicates rows in which the two methods differ by 5% or more.

**Table 4:** Summary of non-mutated residues and corresponding *Q *values.

*Non Mutated Residue*	***Q *value**
Gly 10 (GGC)	0.135*
Trp 11 (TGG)	0.010
Arg 21 (CGA)	0.013
Asn 30 (AAC)	0.004
Ala 31 (GCC)	0.135*
Ser 32 (AGC)	0.020
Pro 52 (CCT)	0.135*
Val 55 (GTC)	0.026
Cys 57 (TGC)	0.021
His 59 (CAC)	0.020
Leu 60 (CTG)	0.058*
Leu 61 (CTG)	0.058*
Lys 63 (AAG)	0.004
His 64 (CAC)	0.020
Ser 67 (TCA)	0.026
Trp 73 (TGG)	0.010
Arg 74 (CGG)	0.164*
Arg 80 (CGG)	0.164*
Glu 84 (GAG)	0.020
Ala 85 (GCC)	0.135*
Tyr 92 (TAC)	0.004
Gly 99 (GGA)	0.135*
Leu 106 (CTG)	0.058*
Ser 108 (TCA)	0.026
Ser 111 (AGC)	0.026
Asp 112 (GAC)	0.020
Cys 113 (TGC)	0.021
Ser 115 (TCA)	0.026
Ala 116 (GCC)	0.135*
Gly 120 (GGA)	0.135*
Leu 122 (CTG)	0.058*
Ala 137 (GCC)	0.135*
Glu 145 (GAG)	0.020
Met 146 (ATG)	0.002
Ser 147 (AGC)	0.026
Val 150 (GTG)	0.026
Gly 155 (GGC)	0.135*
His 157 (CAC)	0.020
Thr 162 (ACT)	0.025

* *Q *value greater than 5%
